# ASPP2 binds to hepatitis C virus NS5A protein via an SH3 domain/PxxP motif-mediated interaction and potentiates infection

**DOI:** 10.1099/jgv.0.001895

**Published:** 2023-09-01

**Authors:** Artem Smirnov, Andrea Magri, Rebecca Lotz, Xiaoyue Han, Chunhong Yin, Mark Harris, Christian Osterburg, Volker Dötsch, Jane A. McKeating, Xin Lu

**Affiliations:** 1 Ludwig Institute for Cancer Research, Nuffield Department of Clinical Medicine, University of Oxford, Oxford OX3 7DQ, UK; 2 Department of Experimental Medicine, TOR, University of Rome “Tor Vergata”, Rome 00133, Italy; 3 Nuffield Department of Clinical Medicine, University of Oxford, Oxford OX3 7FZ, UK; 4 Institute of Biophysical Chemistry and Center for Biomolecular Magnetic Resonance, Goethe University, Frankfurt, Germany; 5 School of Molecular and Cellular Biology, Faculty of Biological Sciences, and Astbury Centre for Structural Molecular Biology, University of Leeds, Leeds, UK; 6 Chinese Academy of Medical Sciences Oxford Institute, University of Oxford, Oxford, UK

**Keywords:** ASPP2, HCV, infection, liver cancer, NS5A, viral replication

## Abstract

Hepatitis C virus (HCV) infects millions of people worldwide and is a leading cause of liver disease. Despite recent advances in antiviral therapies, viral resistance can limit drug efficacy and understanding the mechanisms that confer viral escape is important. We employ an unbiased interactome analysis to discover host binding partners of the HCV non-structural protein 5A (NS5A), a key player in viral replication and assembly. We identify ASPP2, apoptosis-stimulating protein of p53, as a new host co-factor that binds NS5A via its SH3 domain. Importantly, silencing ASPP2 reduces viral replication and spread. Our study uncovers a previously unknown role for ASPP2 to potentiate HCV RNA replication.

## Introduction

Hepatitis C virus (HCV) is a global health problem affecting 58 million people and infection is characterized by an acute phase with mild symptoms that persists to a chronic disease in the majority (55–85%) of cases [1]. Chronic infection is associated with an increased risk of developing liver cirrhosis and hepatocellular carcinoma (HCC) [2]. HCV is a member of the *Flaviviridae* family of viruses with a positive-sense ssRNA genome and infects hepatocytes, whose polarity and tight junctions act as a physical barrier to infection [3, 4]. The viral envelope glycoproteins interact with the tight junction proteins occludin and claudin-1 and transiently perturb hepatocellular polarity to promote viral uptake [4–6]. The HCV genome encodes a single polyprotein that is processed by host and viral proteases to generate ten mature proteins [7]. Direct antiviral agents (DAAs) targeting the non-structural (NS) proteins (NS3-4A, NS5A and NS5B) have revolutionized the treatment of chronic hepatitis C (CHC) with >95% cure rates, but the majority of licensed drugs are optimized for HCV genotype (gt) 1a/b [8, 9]. Resistance-associated substitutions (RAS) in the viral genome have been described for the commonly used DAAs [10], representing a barrier to viral eradication. Of note, multiple resistance-associated substitutions have been identified within the N-terminus of NS5A (gt1) that confer resistance to the NS5A targeting drug daclatasvir [11, 12]. Addressing these clinical challenges requires a greater understanding of how HCV interacts with its host, in particular how viral proteins such as NS5A interact with host factors.

To address this, we used an MS-based proteomics approach to identify the NS5A interactome in the HCV-infected human hepatoma cell line Huh7.5. Interestingly, ASPP2, a member of the evolutionarily conserved ASPP family, was among the top 20 host proteins that interacted with NS5A. The ASPP family consists of three members, ASPP1, ASPP2 and iASPP, which are variously described as ankyrin repeats, SH3 domain, proline-rich sequence-containing proteins, or apoptosis stimulating proteins of p53. ASPP1 and ASPP2 stimulate, whereas iASPP inhibits, the ability of p53 to selectively regulate the expression of pro-apoptotic genes such as Bax [13, 14]. Importantly, ASPPs can shuttle between cell/cell junctions, the cytoplasm and the nucleus [15] and ASPP2 is a binding partner of Par3 that controls apical cell polarity [16] and is a key regulator of cell plasticity [17]. Here we report the biochemical characterization of the ASPP2/NS5A interaction and its potential in influencing HCV biology.

## Methods

### Cell culture and reagents

Huh-7 or Huh-7.5 cells were maintained in DMEM (Life Technologies) supplemented with 10% FBS, 2 mM l-glutamine, 100 U ml^−1^ penicillin, 100 μg ml^−1^ streptomycin and 0.1 mM non-essential amino acids. Huh7-N17 and Huh7.5-N17 cells, stably harbouring the HCV subgenomic replicon N17, were propagated as above with addition of 2 μg ml^−1^ puromycin. Cells were grown in a humidified incubator at 37°C with 5% CO_2_. Cell lines were tested monthly for mycoplasma using a MycoAlert kit (Lonza) or LookOut Mycoplasma PCR detection kit (Sigma-Aldrich). Cell lines used in this study are listed in Table S1, available in the online version of this article.

### Cloning and mutagenesis

To identify NS5A-interacting proteins, a full-length JFH-1 infectious clone was modified by the insertion of a One-Strep Tag (OST) into the C-terminus of NS5A. This was achieved by cloning the corresponding NS5A fragment from SGR-JFH1-5A1ST [18] into the JFH-1 infectious clone [19]. This insertion had no effect on the replicative capacity of the virus (data not shown).

The cDNAs encoding NS5A from the four HCV strains Con1, JFH-1 and ED43, the cDNA of HCV JFH-1 core protein and the cDNAs of the human Fyn, Lyn, Bin1, Lasp1, FGR, Abl1 and Grb2 SH3 domains were obtained with a 5′ *Bam*HI site and a 3′ stop codon and *Xho*I site cloned into a pMX vector (ThermoFisher). ASPP1 and ASPP2 C-terminal domains (CTDs) were amplified by PCR, simultaneously introducing a 5′ *Bam*HI site and a 3′ stop codon (TGA) and *Xho*I site for subsequent restriction cloning. pcDNA3.1(+)-Myc-HCV NS5A and pcDNA3.1(+)-Myc-HCV core plasmids were generated by subcloning the respective cDNAs from the pMX vectors in a pcDNA3.1(+) vector with an N-terminal Myc-tag. For recombinant expression in *Escherichia coli*, inserts of ASPP1 and ASPP2 CTDs were introduced in pET-15b-His10-TEV (N-terminal His10-tag followed by TEV protease cleavage site) and pET-15b-His10-Halo-TEV-3xGS (N-terminal His10-tag followed by Halo-tag, TEV protease cleavage site and GSGSGS linker) by subcloning using the *Bam*HI and *Xho*I restriction sites. Similarly, cDNAs of the other SH3 domains were transferred from the pMX vectors into pGEX-6P-2-His8-TEV (N-terminal GST-tag followed by His8-tag and TEV protease cleavage site) using the *Bam*HI and *Xho*I restriction sites. The pET-15b-His10-Halo-TEV-3xGS vector was generated by subcloning Halo in pET-15b vector using *Nco*I and *Xho*I restriction sites. The His10-tag was engineered into the 5′ oligo and the TEV protease cleavage site, linker, *Bam*HI site and stop codon into by the 3′ oligo. All successive constructs carrying mutations were generated by site-directed mutagenesis. Primers used for subcloning and site-directed mutagenesis are listed in Table S2. pENTR4-HaloTag (w876-1) was a gift from Eric Campeau. Plasmids used in this study are listed in Table S1.

### Strep-tagged NS5A pulldown and MS

*In vitro* transcribed JFH1 RNA in which NS5A was tagged with an OST (JFH-1-NS5A-OST) was electroporated into Huh7.5 cells and seeded in 10×10 cm dishes. Untagged JFH-1 RNA was used as a negative control. Cells were washed twice in PBS 72 h after electroporation, pelleted at 1500 ***g*** for 5 min at room temperature (RT), before lysis in GL buffer (10 mM PIPES/NaOH pH 7.2, 120 mM KCl, 30 mM NaCl, 5 mM MgCl_2_, 1% Triton X100, 10% glycerol). Lysates were clarified by centrifugation at 10 000 ***g*** for 5 min at 4 °C. Supernatants were applied to 100 μl of Strep-Tactin Sepharose for batch affinity purification of NS5A-OST following the manufacturer’s instructions. After overnight incubation at 4 °C, the mixtures were washed three times with 100 mM Tris/HCl, pH 8.0, 150 mM NaCl and 1 mM EDTA. Bound protein was eluted with SDS-sample buffer. For multiplexed comparative proteomics each eluate was digested with trypsin and labelled with tandem mass tag (TMT) reagents according to the manufacturer’s protocol (Thermo Fisher Scientific). The labelled samples were then pooled, evaporated to dryness and resuspended prior to fractionation by high pH reversed-phase chromatography using an Ultimate 3000 liquid chromatography system (Thermo Fisher Scientific). After high pH reversed-phase chromatography, fractions were further subjected to Nano-LC MS. The raw data files were processed and quantified using Proteome Discoverer software v1.4 (Thermo Scientific) and searched against the Uniporter Human database (134169 sequences) plus HCV protein sequences using the SEQUEST algorithm. All peptide data were filtered to satisfy a false discovery rate (FDR) of 1%. MS mass tolerance was 20 p.p.m., and fragment ion mass tolerance was 0.05 Da. For all proteins, an abundancy score was calculated as the ratio of abundancy in HCV-TST versus TST-only samples. Proteins from the 10% percentile (abundancy >1.4) were considered as hits. Identified hits are listed in Table S3.

### Protein expression and purification

All expression plasmids were transformed in the *E. coli* strain Rosetta DE3 (Merck Millipore) for protein production. Cells were grown in 2×YT medium to an OD of ~0.8. Expression was carried out for ~18 h at either 16 °C for ASPP CTDs or at 22 °C for the other proteins. Cells were harvested, resuspended in ice-cold IMAC A buffer [25 mM Tris pH 7.8, 200 mM NaCl, 20 mM *β*-mercaptoethanol (*β*-ME), 5% glycerol, 25 mM imidazole] supplemented with lysozyme (Sigma-Aldrich), RNAse (Sigma-Aldrich), DNAse (Sigma-Aldrich) and protease inhibitors (Carl Roth) and lysed by sonification. Lysate was cleared by centrifugation at 4 °C. All subsequent purification steps were performed at 4 °C using an ÄKTA Purifier chromatography system (Cytiva). As the first purification step all proteins were purified by Ni^2+^-affinity chromatography using HisTrap columns (Cytiva). Halo fusion proteins were further purified by ion-exchange (IEX) chromatography. The salt concentration was reduced below 100 mM by dilution with IEX A buffer (25 mM Tris pH 7.8, 50 mM NaCl, 20 mM *β*-ME, 5% glycerol) prior loading on HiTrap Q columns (Cytiva). Bound protein was eluted with a gradient from 50 to 1000 mM NaCl over 20 column volumes using IEX B buffer (25 mM Tris pH 7.8, 1000 mM NaCl, 20 mM *β*-ME, 5% glycerol). Finally, proteins were subjected to size exclusion chromatography (SEC) with a Superdex 200 column (Cytiva) equilibrated in the storage buffer (25 mM HEPES pH 7.5, 150 mM NaCl, 0.5 mM TCEP (Tris(2-carboxyethyl)phosphine hydrochloride), 10% glycerol). His-tagged ASPP CTDs and GST-His-tagged SH3 domains were instead supplemented with His-tagged TEV protease and dialysed overnight at 4 °C in IMAC A buffer to cleave off the tag and remove excess imidazole simultaneously. A reverse Ni^2+^-affinity purification as a second purification step removed the tag and TEV protease. The flow-through containing the cleaved protein was further purified by IEX chromatography as described for the Halo fusion proteins. In a final polishing step, the protein was loaded on a Superdex 75 s column (Cytiva) equilibrated in the storage buffer (25 mM HEPES pH 7.5, 150 mM NaCl, 0.5 mM TCEP). All proteins were concentrated by centrifugation, snap frozen in liquid nitrogen and stored at −80 °C until usage. Plasmids used in this study are listed in Table S1.

### Isothermal titration calorimetry

All isothermal titration calorimetry (ITC) experiments were performed on a VP-ITC device (MicroCal). A tryptophane residue was added to the N-terminus of all peptides for accurate determination of the concentration via absorption at 280 nm. Dissolved peptides and purified proteins were dialysed in ITC buffer (25 mM HEPES pH 7.5, 150 mM NaCl, 500 μM TCEP) overnight at 4 °C. All measurements were performed with 25 titration steps of 10 μl each. Then 450 or 300 μM NS5A peptides were titrated to 30 μM ASPP family CTDs and SH3 domains at 12 °C. As a control, 200 μM of a peptide without proline-rich region (PRR) was titrated to 13 μM ASPP2 CTD. Binding curves were analysed using NITPIC [20, 21] including subtraction of dilution heat measurement of each ligand. The thermodynamic parameters (Δ*H* and *T*Δ*S*), the equilibrium dissociation constant (*K*_D_) and the incompetent fractions of A (incfA) and B (incfB) were determined with SEDPHAT [22] assuming an AB hetero association model. Final ITC figures were generated by GUSSI [18]. ITC values are listed in Table S4.

### Halo pull-down

One hundred micrograms of purified Halo or Halo-tagged ASPP family CTDs (ASPP1: 887–1090, ASPP2:925–1128) were covalently immobilized on 25 μl magnetic Halo beads slurry in pulldown buffer (50 mM HEPES pH 7.5, 200 mM NaCl, 0.5 mM TCEP, 0.1% Surfactant P-20) overnight at 4 °C. The following day, myc-tagged NS5A wild-type and mutants of the indicated HCV strains or HCV core protein were produced by *in vitro* translation (Promega; L1170) from pcDNA3.1(+) plasmids. Reactions were incubated for 90 min at 30 °C. DNA and RNA was digested by using benzonase nuclease for 30 min at 4 °C and reactions were cleared by centrifugation for 10 min at 13 000 ***g*** to remove aggregates. Loaded beads were washed three times with pull-down buffer to remove unbound Halo proteins and incubated with 20 μl *in vitro* translated protein and 10 μM dissolved peptide, if indicated. Following rotation for 3 h at 4 °C, beads were washed four times with ice-cold pulldown buffer and bound proteins were eluted in 1× LDS sample buffer (Thermo Fisher) supplemented with 0.5 mM DTT for 10 min at 70 °C. Input and pulldown samples were analysed by Western blot using anti-myc tag antibody (Merck Millipore) and anti-Halo tag antibody (Promega).

### Co-immunoprecipitation

For exogenous co-immunoprecipitation, N-terminally myc-tagged ASPP2 and C-terminally HA-tagged NS5A (WT or PRR mutant) were transfected into H1299 cells for 24 h. For co-immunoprecipitation in hepatoma cells, Huh-7, Huh-7.5, Huh-7-N17 and Huh-7.5-N17 cells were grown in T175 flasks. Cells were lysed in RIPA lysis buffer (50 mM HEPES pH 7.5, 150 mM NaCl, 1% NP-40, 1% sodium deoxycholate, 1 mM DTT, 1 mM MgCl_2_) supplemented with phosphatase and protease inhibitors. DNA and RNA was digested by using benzonase nuclease for 30 min at 4 °C. Then 10 mg of cell lysate was incubated with 6 μg of anti-NS5A, anti-ASPP2 or IgG control antibodies to carry out each co-immunoprecipitation experiment. Lysates were cleared by centrifugation for 15 min at 13 000 ***g*** and supernatant was incubated with primary antibody overnight at 4 °C. Immunocomplexes were purified using Protein G Dynabeads (Thermo Fisher Scientific) for 3 h at 4 °C (exogenous IP) or 1 h at RT (endogenous IP). Beads were washed four times with ice-cold IP buffer (50 mM HEPES pH 7.5, 200 mM NaCl, 0.1% Surfactant P-20) and bound proteins were eluted in 1×LDS sample buffer (Thermo Fisher) supplemented with 0.5 mM DTT for 10 min at 70 °C. Input and pulldown samples were analysed by Western blot. Antibodies used in this study are listed in Table S1.

### RNA interference

For knock-down experiments, cells were transfected with small interfering RNA (siRNA) using Lipofectamine RNAiMAX (Invitrogen) for 48 h. For each gene, a pool of four siRNAs was used. siRNAs were synthesized by Merck. siRNA target sequences used in this study are listed in Table S2.

### Western blot

Cell pellets were lysed with 8 M urea buffer on ice for 30 min and then lysates were cleared by centrifugation at 13 000 ***g*** for 20 min at 4 °C. A Bradford protein assay (BioRad) was used to measure protein concentration. Proteins were separated on SDS-PAGE columns and transferred onto PVDF membranes (BioRad), blocked at RT for 1 h in 10% non-fat dry milk in TBS buffer with 0.1% Tween-20 (TBST) and incubated with primary antibodies overnight at 4 °C. Membranes were then washed for 30 min in TBST, incubated with HRP-coupled secondary antibodies (Dako) at 1:1000 dilution in TBST at RT for 1 h, and visualized with ECL Prime Western Blotting System reagents. Antibodies used in this study are listed in Table S1.

### RNA extraction and qPCR

Total RNA was extracted from cells with the RNeasy Mini Kit (Qiagen) following the manufacturer’s protocol. For cDNA conversion, 500 ng of total RNA sample was reverse transcribed using the qPCRBIO cDNA Synthesis Kit (PCR Biosystems) with a mixture of oligo(dT) and random hexamers. Quantitative PCR (qPCR) was performed on cDNA samples using the qPCRBIO SyGreen Blue Mix (PCR Biosystems) on the Lightcycler 96 (Roche). Each qPCR was carried out in duplicate. The expression level of target genes was analysed using the comparative Ct method (ΔΔCt) with *RPLP0* as the internal housekeeping gene. For absolute quantification of viral copies, a standard curve was generated using a 1:10 serial dilution of known copies of a plasmid containing an HCV genome. Primers used for the analysis are listed in Table S2.

### Immunofluorescence and microscopy

Cells were seeded on collagen-coated coverslips (for confocal microscopy) or in 96-well plates (for automated quantification by Operetta). For immunofluorescence staining, cells were fixed in 4% methanol-free paraformaldehyde solution in PBS for >10 min, rinsed with PBS, permeabilized with 0.25% Triton X-100 in PBS for 10 min at RT and blocked with 10% BSA solution in 0.1% PBS-Tween-20 (PBST) for 2 h at RT. Cells were stained with primary antibodies diluted in blocking buffer overnight at 4 °C. The next day, the cells were washed three times with PBST buffer and then incubated with 2 μg ml^−1^ of secondary antibody and 1 μg ml^−1^ Hoechst 33342 for 1 h at RT. After three washes with PBST, plates were filled with PBS, while coverslips were mounted into slides using ProLong Gold Antifade Mountant (ThermoFisher). NS5A subcellular localization and expression was analysed using an LSM 980 confocal microscope (Zeiss) in super-resolution mode. Images were produced using Zen3.0 software (Zeiss). For automated quantification of HCV-positive cells and foci properties, samples were analysed using the Operetta high-throughput screening system (PerkinElmer) and Harmony software (PerkinElmer). In brief, a threshold for identification of HCV-NS5A-positive cells was set as a [mean+2×(sd)] value of fluorescence of control uninfected cells. Foci were detected with a maximum distance of 20 μm between two positive cells and foci properties were calculated. Antibodies used in this study are listed in Table S1.

### HCV stock production and infection

HCV strain J6/JFH-1 [23] was used in this study. Briefly, *in vitro* transcribed RNA was electroporated into Huh-7.5 cells using Gene Pulser Xcell (Bio-Rad) as previously reported [24]. Supernatant from the transfected cells was collected 72 h post-electroporation and titrated on naïve Huh-7.5 cells. For infection experiments, 48 h after ASPP2 knock-down, Huh-7 or Huh-7.5 cells were infected with HCV for 3 h, the inoculum was removed and cells were supplemented with fresh media. After 48 h the supernatants were collected for analysis of extracellular viral RNA by reverse transciption (RT)-qPCR, while cells were subjected to Western blot, RT-qPCR or immunofluorescence analysis.

### HCV replication

HCV stable cell lines were generated as previously reported [25]. Briefly, the plasmid containing the monocistronic replicon N17/JFH was linearized using *Mlu*I (NEB). After purification, the linear DNA was used as template to produce RNA using a T7 *in vitro* transcription kit (Life Technologies). The RNA was electroporated into Huh-7 or Huh-7.5 cells and 24 h later the cells were selected with puromycin (2 μg ml^−1^) until colonies formed. Clones were then trypsinized and pooled to generate polyclonal lines.

### Plasmid transfection

pNL4.3^E-R-^ plasmid was transfected into Huh-7.5 cells using PEI (Polysciences) following the manufacturer’s protocol. Briefly, DNA was diluted in Optimem with an appropriate amount of PEI and incubated at RT for 15 min before being added to the cells. Four hours later, media were replaced with fresh complete DMEM and incubated as appropriate.

### HCV and VSVpp production

HCV and vesicular stomatitis virus (VSVpp) were generated as previously reported [26]. Briefly, pNL4.3.Luc.E^-^R^-^ and plasmids encoding HCV E1E2 or VSV-G glycoproteins were co-transfected in HEK293T using PEI as described above. Forty-eight hours after transfection, supernatants were collected, filtered, aliquoted and stored at −80 °C. To test viral entry, 48 h after knock-down (KD), Huh-7.5 cells were inoculated with HCVpp or VSVpp for 3 h. After infection, the inoculum is removed, cells washed with PBS once and fresh media added. After 48 h, cells were lysed, transferred to a 96-well white microplate and luciferase measured as described above.

### Statistical analyses

All analyses were performed in GraphPad Prism 9 (GraphPad). ImageJ was used to conduct densitometric analysis of NS5A expression. One-way ANOVA was used to determine the significance of differences between two groups. *P*<0.05 was considered significant.

## Results

### HCV NS5A interactome identifies new binding partners

**Fig. 1:**
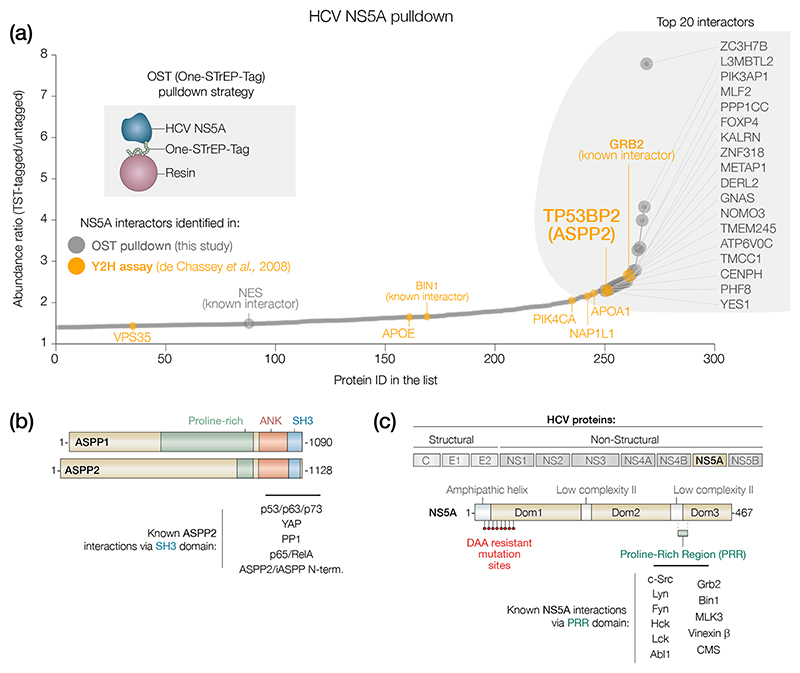
HCV NS5A interactome identifies new binding partners. (a) Dot plot showing the distribution of identified host co-factors of HCV NS5A by OST pulldown followed by MS. Data are shown as abundance enrichment of proteins in the Strep-tagged sample over untagged control. Proteins from yeast two-hybrid (Y2H) assay [31] are highlighted in orange. The top 20 interactors are labelled on the plot. (b) Schematic representation of the structure of ASPP1 and ASPP2 proteins. Proline-rich: proline-rich domain, ANK: ankyrin repeats, SH3: SH3 domain. (c) Schematic diagram of the HCV genome (top panel) and NS5A protein structure (lower panel). C: Core, E1/E2: envelope glycoproteins, NS25B: non-structural proteins. DAA resistance-associated substitutions are highlighted in red. The location of the proline-rich region in NS5A is labelled as PRR to distinguish it from the proline-rich region in ASPPs and is shown in green.

To identify potential host factors that regulate HCV replication, Huh-7 cells were electroporated with JFH-1-NS5A-OST RNA (a JFH-1 derivative in which NS5A was tagged with the OST in domain III [27]). Immunoprecipitation of NS5A and MS identified 269 putative co-factors that specifically bound to the tagged NS5A compared with an untagged NS5A control (abundancy enrichment >1.4) (Fig. 1a, Fig. S1, Table S3). We were able to identify previously described interactors such as PI4KIIIa [28], Bin1 [29] and Grb2 [30], suggesting the experimental conditions used are suitable for NS5A interactome discovery. Interestingly, among the top 20 interactors we found ASPP2 (encoded by the *TP53BP2* gene) as an interacting partner of NS5A. ASPP2 was among 340 proteins previously reported as an NS5A binding partner in a yeast two-hybrid assay [31], but its role in influencing HCV function remains unknown. Therefore, we focused on ASPP2 and investigated its potential as a key host interactor of NS5A.

### ASPP2 interacts with HCV NS5A via SH3/PxφPx+ domains

**Fig. 2:**
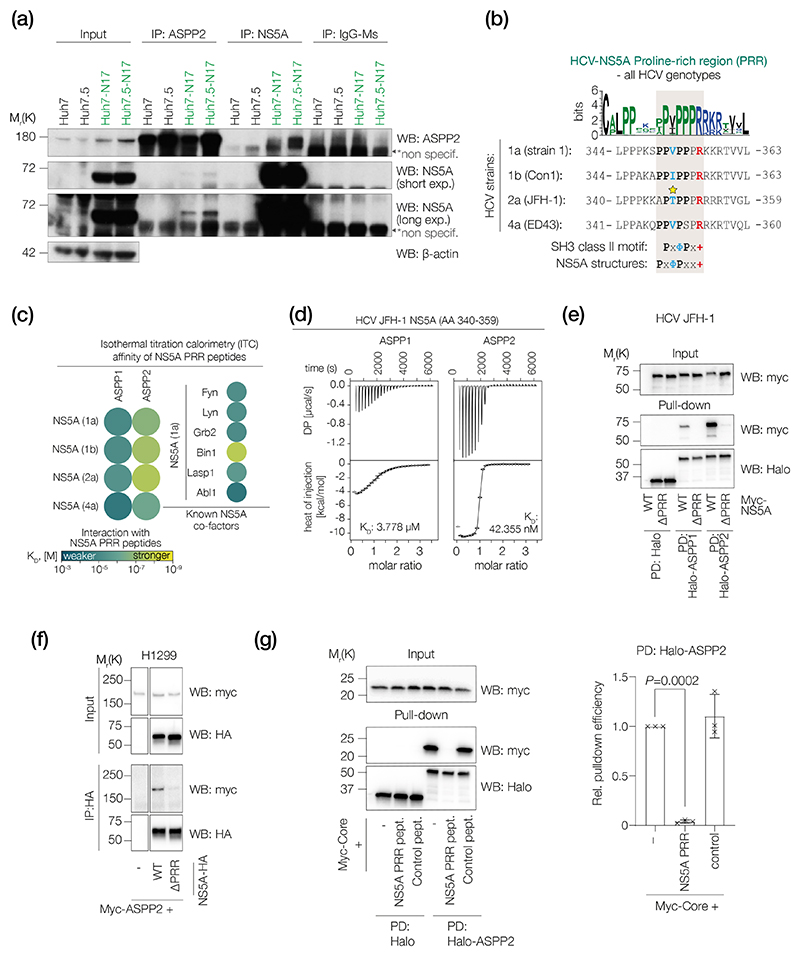
ASPP2 interacts with HCV NS5A via its SH3 domain. (a) Western blot analysis of ASPP2 and NS5A co-immunoprecipitation in parental Huh7 and Huh7.5 or N17-replicon expressing Huh7-N17 and Huh7.5-N17 cells. Antibodies against ASPP2, NS5A or irrelevant mouse IgG were used for immunoprecipitation (IP). (b) Sequence alignment of the predicted SH3 motifs of NS5A from the indicated HCV strains with the canonical SH3 class II motif and the atypical class II motif revealed by the Fyn NS5A complex structure (PDB: 3UA7, see [47]). Essential residues are in bold and colour coded (ϕ: any aliphatic residue; +: any positively charged residue). The reported phosphorylation site is marked by a star [70]. The consensus sequence of the NS5A PRR is shown above. (c) Heatmap representation of NS5A PRR peptide binding affinities for ASPP1 and ASPP2 CTDs and other SH3 domains measured by ITC. Detailed results are shown in Fig. S2B. (d) Representative ITC curves from (c). (e) Western blot analysis of pull-down assay using immobilized recombinant Halo-tagged ASPP family CTDs to pull down *in vitro* translated Myc-tagged NS5A WT and ΔPRR (Δ340–359) mutant from HCV genotype 2a strain JFH-1; *n*=3 (biological replicates). Anti-myc and anti-Halo antibodies were used to detect NS5A or ASPPs, respectively. PD: pull-down. (f) Western blot analysis of co-immunoprecipitation using full-length Myc-tagged ASPP2 and HA-tagged NS5A [JFH-1 strain, WT or ΔPRR (Δ340–359) mutant] transiently transfected into H1299 cells. NS5A was immunoprecipitated (IP) with an anti-HA antibody and anti-HA and anti-Myc antibodies were used to detect NS5A or ASPP2, respectively. (g) (Left) Western blot analysis of pull-down assay using immobilized recombinant Halo-tagged ASPP2 CTDs to pull down *in vitro* translated Myc-tagged Core in the absence or presence of NS5A PRR peptide (JFH-1) or a control peptide lacking a PRR. Anti-myc and anti-Halo antibodies were used to detect Core or ASPP2, respectively. (Right) Relative pull-down efficiency is shown as mean±sd; *n*=3 (biological replicates). *P*-value by one-way ANOVA test. PD: pull-down.

To validate the observed ASPP2-NS5A binding, we generated Huh7 and Huh7.5 cell lines stably harbouring the monocistronic subgenomic replicon N17/JFH (Huh7-N17 and Huh7.5-N17 respectively). As shown in Fig. 2a), anti-ASPP2 immunoprecipitates from both Huh7-N17 and Huh7.5-N17 cell lysates contained NS5A. In contrast, an anti-NS5A antibody also co-immunoprecipitated endogenous ASPP2 from both Huh7-N17 and Huh7.5-N17 cell lysates. Interestingly the amount of ASPP2-NS5A complex detected in Huh7.5-N17 cells was higher than that in Huh7-N17 cells, consistent with the endogenous ASPP2 level being higher in Huh7.5-N17 than that in Huh7-N17 cells (Fig. 2a). This result suggests that ASPP2 might be a key host interactor of NS5A in cells.

ASPP2 and the related ASPP1 share high sequence similarity whereas iASPP has a different N-terminal sequence [32, 33]. ASPP1 and ASPP2 are known to bind an SH3 class II binding motif, PxϕPx+ via their C-terminal domains (Fig. 1b) [34–40]. The PRR between the low complexity sequence 2 (LCSII) and Domain 3 of NS5A contains a PxϕPx+ motif and mediates the interaction with several SH3 domain-containing host proteins that have been reported to regulate NS5A phosphorylation and viral replication [30, 41–46] (Fig. 1c). We hypothesized that the SH3 domain of ASPP2 binds an NS5A PRR and evaluated this hypothesis using both computational and biochemical analyses.

Analysis of complete HCV genome sequences published in the NCBI database identified a PRR motif in the majority (200/202) of viral strains (Table S2) that was conserved across diverse HCV gts (Figs 2b and S2a). The crystal structures of HCV strain Con1 (gt1b) PRR (APPIPPPR) bound to the SH3 domains of c-Src, Fyn and MLK3 revealed two conformations, PxϕPx+ and PxϕPxx+ [47–49]. The canonical SH3 class II motif, PxϕPx+, is conserved in all HCV PRRs, with the exception of strain ED43 (gt4) and strain EUH1480 (gt5), which contain the related PxϕPxx+ motif (Fig. 2b and Table S5).

To determine whether the HCV NS5A PRR could interact with ASPP2, we synthesized peptides of 20 aa in length from the NS5A PRR of four HCV strains (sequences in Fig. 2b) and measured their interaction with a recombinant ASPP2 CTD by ITC (Figs 2c, d and S2b, Table S3). ASPP2 bound all of the NS5A PRRs with high affinity in the nanomolar range. Under the same conditions, the CTD of ASPP1 showed only moderate binding. A control peptide (GSHEFSSPSHLLRTPSSASAVAVGSSETRGERW) lacking a PRR motif showed no interaction with ASPP2 (Fig. S2c). The affinity of ASPP2 CTD binding to HCV JFH-1 NS5A PRR was 10–20 times higher than that of ED43 PRR, suggesting the ASPP2 CTD preferentially binds the canonical PxϕPx+ overthe PxϕPxx+ motif (Fig. 2c). Measuring HCV gt1a NS5A PRR affinity for the SH3 domains of six known cellular binding partners identified Bin1 [29] as having the highest binding affinity for NS5A. ASPP2 and Bin1 show similar binding affinities for NS5A, whereas all other previously characterized NS5A binding proteins including GRB2 [30], Fyn, Lyn[43], Lasp1 [42] and Abl1 [50] bound with lower affinity. *In vitro* translation of full-length NS5A using a rabbit reticulocyte-based cell-free expression system confirmed binding with ASPP2 CTD. A recombinant Halo-tagged ASPP2 CTD bound NS5A wild-type but not a ΔPRR mutant, while no interaction was detected for Halo-tagged ASPP1 CTD or the isolated HaloTag (Figs 2e and S2d). The observed differences in pull-down efficiency are consistent with the affinities measured by ITC.

To confirm that the identified PRR region of NS5A is required to mediate ASPP2-NS5A binding in cells and that this interaction is not specific to hepatocytes nor dependent on p53, the originally identified ASPP2 binding partner, we co-transfected an myc-tagged ASPP2 with HA-tagged wild-type full-length NS5A or the PRR-deleted derivative NS5A-ΔPRR into the p53 null human lung cancer cell line H1299. HA-tagged NS5A was immunoprecipitated by an anti-HA antibody and the presence of ASPP2 in the co-immunoprecipitates was detected by an antibody that recognizes the myc tag. The results in Fig. 2(f) showed that only wild-type full-length NS5A but not NS5A-ΔPRR can bind co-transfected ASPP2, confirming the requirement of the NS5A PRR region to mediate ASPP2-NS5A binding in H1299 cells.

The ASPP2 CTD was previously reported to interact with the N-terminal 46 aa of HCV encoded Core protein, which lacks a detectable PRR domain [51]. Using a similar pull-down protocol, we showed a Core (genotype 2a strain JFH-1) interaction with ASPP2 which was significantly reduced in the presence of the NS5A PRR peptide (also from JFH-1) but not in the presence of a control peptide missing a PRR (Fig. 2g), suggesting that NS5A and Core are competing for the same ASPP2 binding site. In summary, our *in vitro* studies show that HCV NS5A binds ASPP2, and this is mediated by a conserved PRR domain that interacts with the C-terminus of ASPP2.

### ASPP2 protein levels are associated with HCV replication potential

**Fig. 3:**
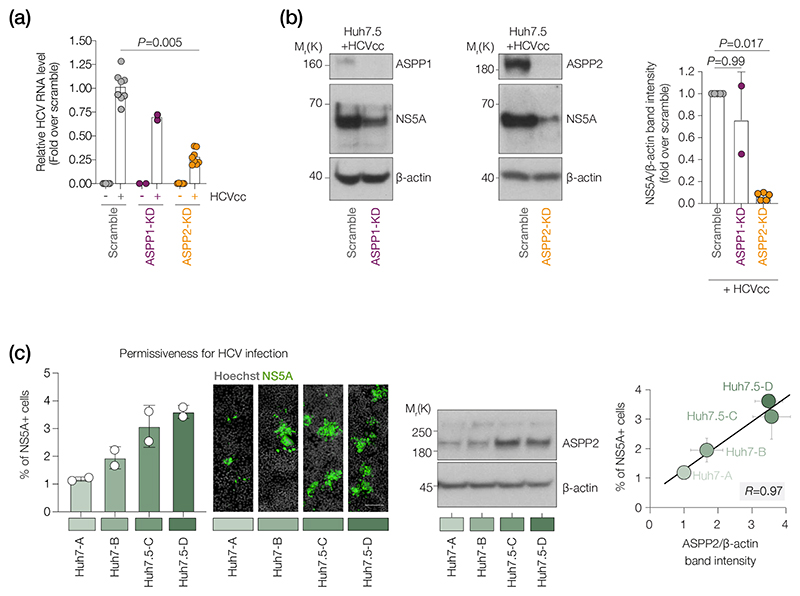
ASPP2 promotes HCV infection of Huh-7 hepatoma cells. (a) RT-qPCR analysis of normalized intracellular HCV RNA levels in scramble, ASPP1-KD and ASPP2-KD samples at an m.o.i. of 1. Data are presented as mean±sd. Individual values are shown. *P*-values by one-way ANOVA test (compared with scramble); *n*=1 (ASPP1-KD) or *n*=4 (ASPP2-KD) biological replicates with two technical replicates each. (b) (Left and middle) Western blot analysis of ASPP1, ASPP2 and NS5A expression in Huh-7.5 cells after HCV infection. *β*-Actin is shown as a loading control. (Right) Densitometric analysis of normalized NS5A expression levels. Data are presented as mean±sd with individual values shown. *P*-values by one-way ANOVA test (compared with Si scramble); *n*=5 (ASPP2 KD) or *n*=2 (ASPP1 KD) biological replicates. (c) (Left) Percentage of NS5A-positive cells in indicated cell lines after HCV infection. Representative images are shown on the right; *n*=1 biological replicate with two technical replicates. (Centre) Western blot analysis of ASPP2 expression in indicated cell lines with *β*-actin as a loading control; *n*=2 (biological replicates). (Right) Pearson correlation (*r*) analysis between percentage of NS5A-positive cells and ASPP2 expression.

To assess the biological consequences of ASPP2 binding to NS5A we silenced its expression in Huh-7.5 hepatoma cells [52] and quantified susceptibility to HCV infection. HCV (strain J6/JFH1) replication was assessed by quantifying intracellular RNA or NS5A expression. ASPP2 KD led to a significant decrease in HCV RNA levels (>80%) and NS5A expression (95%). ASPP1 KD had a modest effect on HCV replication (40–50%) (Fig. 3a, b), consistent with its lower binding affinity for NS5A. Reduced ASPP2 expression in parental Huh-7 cells also reduced NS5A expression (Fig. S3a).

To extend this analysis, we measured endogenous ASPP2 expression in two independently sourced Huh-7 cell lines (labelled A and B) and one Huh-7.5 cell line (labelled as C) in addition to the Huh-7.5 cells used in Fig. 3(a, b) (labelled as D). As previously reported, Huh-7-derived cell lines support variable levels of HCV replication [53, 54] and we observed an association between ASPP2 expression and susceptibility to HCV infection (Fig. 3c). In summary, these data illustrate that ASPP2 expression levels are positively associated with HCV infection potential.

### ASPP2 potentiates HCV RNA replication

**Fig. 4:**
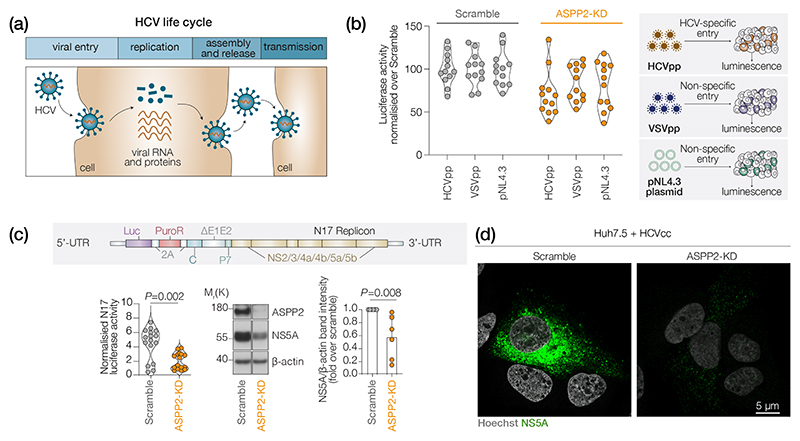
ASPP2 regulates HCV RNA replication. (a) Schematic illustration of the HCV life cycle. (b) Normalized luciferase activity of HCVpp-or VSVpp-infected or pNL4.3 vector-transfected Huh-7.5 cells. The pseudo-particle experiment was performed by infecting Huh-7.5 cells with three different inoculations, with four technical replicates per infection. In parallel, Huh-7.5 cells were transfected with three quantities of pNL4.3^E-L-^ plasmid, with four technical replicates per condition. Data are presented as mean values normalized over Scramble ±sd. (c) (Top) Schematic illustration of the N17-replicon construct. (Bottom left) Normalized luciferase activity in replicon Huh-7.5-N17 cells 96 h after ASPP2 KD; *n*=6 biological replicates with four technical replicates each. Western blot and band densitometry analysis of ASPP2 and NS5A expression in the same samples. *β*-Actin is shown as a loading control. (d) Representative confocal micrograph of NS5A staining in Scramble or ASPP2 KD Huh-7.5 cells; bar, 5 μm.

To understand whether ASPP2 affects the HCV infection cycle (Fig. 4a), we first examined whether ASPP2 influences viral entry using lentiviral pseudoparticles (pp) containing the HCV envelope glycoproteins (HCVpp). HCVpp have been widely used to study receptor-dependent entry into hepatocytes [26]. As a control, we generated pp-expressing encoded glycoprotein of VSV, a prototypical Rhabdovirus that displays broad tissue tropism. We noted a modest 2-fold reduction in both HCVpp and VSVpp infection of ASPP2 KD cells compared to the control scramble siRNA-treated cells (Fig. 4b), suggesting a non-viral-specific reduction in lentivirus reporter expression. Moreover, delivering the lentivirus plasmid directly into cells and bypassing the host receptors showed a similar reduction in reporter activity in the ASPP2 KD cells (Fig. 4b). These data suggest that ASPP2 has a minimal effect on the HCV-specific cellular entry process.

To study viral RNA replication we used Huh-7.5 cells stably harbouring a monocistronic HCV replicon N17 that encodes a luciferase reporter gene (Fig. 4c) [25]. ASPP2 KD significantly reduced viral replication as measured by luciferase activity and NS5A expression (Fig. 4c). Real-time monitoring of HCV-driven luciferase activity showed a time-dependent reduction in the ASPP2 KD cells compared to control scrambled siRNA-treated cells (Fig. 4c). We confirmed a similar phenotype in parental Huh-7 cells (Fig. S4a). To understand whether ASPP2 KD affects NS5A localization we imaged the replicon harbouring cells by immunofluorescence microscopy (Fig. 4d). Consistent with the data in Fig. 4(c), we observed lower levels of cytoplasmic NS5A in the ASPP2 KD cells; however, the residual NS5A staining was insufficient to assess any changes in cellular distribution.

As NS5A and NS5B are components of the replication machinery and targets of DAAs, we evaluated the potential role of ASPP2 KD to influence drug efficacy. Treating Huh-7.5 cells harbouring the N17 replicon with drugs targeting NS5A (Daclatasvir) or NS5B (Sofosbuvir) showed a dose-dependent inhibition of HCV replication (Fig. S4b). As expected, the reduced level of HCV RNA in the ASPP2 KD cells resulted in a modest yet significant increase in sensitivity to both DAAs, as determined by the concentration required to inhibit 50% of viral replication (IC_50_). Under the same conditions, ASPP1 KD did not affect the activity of the tested DAAs. Together, our data show that ASPP2 positively influences HCV RNA replication.

### ASPP2 facilitates HCV transmission

**Fig. 5:**
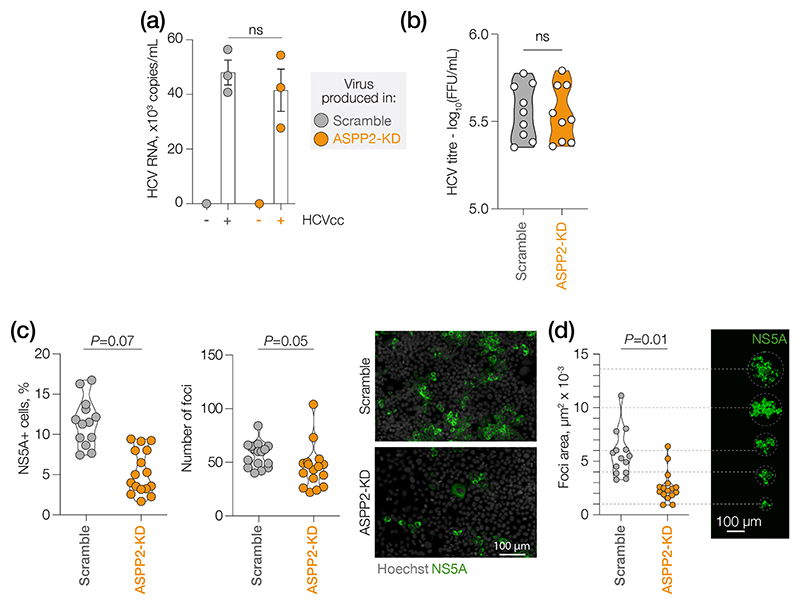
ASPP2 regulates HCV cell–cell spread. (a) qPCR analysis of secreted viral RNA (copies ml^–1^) from scramble or ASPP2 KD samples infected with HCV at an m.o.i.=1. Data are presented as mean±sd. Individual values are shown. *P*-values by one-way ANOVA test (compared with scramble); *n*=3 biological replicates. (b) Viral titres in naïve Huh-7.5 cells of scramble or ASPP2 KD samples after infection with secreted virus. Data are presented as mean values, with individual values shown. *P*-values by one-way ANOVA test (compared with scramble); *n*=3 biological replicates. (c) (Left) Percentage of NS5A-positive cells and number of identified foci in scramble or ASPP2 KD samples after infection with virus at an m.o.i.=0.4. Data are presented as violin plots. Individual values are shown. *P*-values by one-way ANOVA test (compared with scramble); *n*=4 biological replicates with four technical replicates each. (Right) Representative confocal micrographs; bar, 100 μm. (d) (Left) Mean area of foci in the same samples. Data are presented as violin plots. Individual values are shown. *P*-values by one-way ANOVA test (compared with scramble); *n*=4 biological replicates with four technical replicates each. (Right) Representative confocal micrographs of corresponding foci; bar, 100 μm.

HCV can transmit via the release of extracellular particles that infect new target cells or through cell–cell contacts [55]. Both routes of infection require expression of the receptor complex (CD81, scavenger receptor BI, claudin-1 and occludin). As claudin-1 and occludin are part of the tight junction and adhesion complex [56] and ASPP2 maintains apical polarity/tight junction integrity and cell–cell adhesion [16, 17], we were interested to investigate whether ASPP2 KD may influence HCV infection: secretion of virus, cell-to-cell spread or both. We noted that ASPP2 KD cells showed a modest reduction in the levels of secreted HCV RNA, while there were no changes in the amount of infectious virus compared to the scramble control (Fig. 5a, b). To assess the impact of ASPP2 on HCV spread from primary infected cells to neighbouring cells, we imaged NS5A and quantified the frequency of antigen-expressing cells and their location within infection foci (Fig. S5). Although ASPP2 KD reduced the total number of NS5A^+^ cells, the count of foci was comparable to that of the control cells (Fig. 5c, left panel). Notably, however, ASPP2 KD had a significant impact on the size of foci (Fig. 5c, right panel). The mean size of foci in the scramble controls ranged from 4 to 11×10^3^ μm^2^, while the mean size in the KD cells was lower (1–6×10^3^ μm^2^) (Fig. 5d). Together, these findings are consistent with a model in which ASPP2 may play a positive role in controlling HCV spread to neighbouring cells.

## Discussion

Here, we report the identification of ASPP2 as a positive regulator of HCV infection and a key host cellular binding partner of the HCV NS5A protein, an interaction mediated via the ASPP2 SH3 domains and a PxϕPx+ motif within NS5A. ASPPs were originally identified as binding proteins of p53, p65, Bcl2 and PP1 [57] in Y2H assays via their C-terminus, where all three protein binding domains are located. In addition to binding p53 family members, an increasing number of ASPP interacting proteins have been reported and many are involved in various signalling and tissue homeostasis roles, including YAP, Ras and Par3 [16, 58, 59]. Interestingly, among all three ASPP family members, ASPP2 is the one most closely associated with infection. First, ASPP2 is a transcriptional target of STAT1, a transcription factor that is activated upon infection [60]. Second, ASPP2 was identified as a key binding partner of CagA, a toxin of *Helicobacter pylori*, and the CagA/ASPP2 interaction disrupts gastric epithelial polarity and facilitates colonization of CagA+ *H.pylori* [61, 62]. Third, ASPP2 was identified as a direct cellular binding partner of HCV Core. The N-terminal 46 aa portion of the Core protein was reported to bind the ankyrin repeats and SH3 domain of ASPP2, thereby preventing ASPP2 from binding and stimulating p53-mediated apoptosis [51]. Lastly, using an unbiased MS approach (this study) and Y2H assay [31], ASPP2 has emerged as a cellular binding partner of HCV NS5A, emphasizing the biological importance of ASPP2 as a cellular gatekeeper of infection.

NS5A has the potential to modulate other host signalling pathways via the PRR, an interaction hub for a multitude of SH3 domain-containing signalling molecules such as Src and Grb2. The c-Src kinase family activity [43, 50] and Grb2-dependent suppression of EGF-induced MAPK signalling [30, 63] and inhibition of Bin1- and MLK3-induced apoptosis [41, 45] play key roles in HCV replication. Notably, NS5A function is regulated by Ser/Thr and tyrosine phosphorylation [64]. Specifically, NS5A phosphorylation is regulated by Abl1 [65] and our ITC results provide insights into how tyrosine phosphorylation may be regulated via PRR and SH3 domain interactions. Although ASPP2 is a known binding partner of PP1, it is unclear whether the identified NS5A–ASPP2 complex may influence Ser/Thr NS5A phosphorylation status as PP1 and NS5A bind to the same region of ASPP2 with similar binding affinity *in vitro*.

The NS5A PRRs from different HCV strains bound to the ASPP2 CTD with nanomolar affinities, while ASPP1 CTD bound with micromolar affinity. Notably, the NS5A PRR of the Con1 and JFH-1 strains bound the ASPP2 SH3 domain with a greater affinity than PP1α (140 nM), the strongest known binding partner of the ASPP2 SH3 domain reported to date [40]. The interactions of ASPP2 CTD with the p53 family members and PP1 are primarily mediated by the SH3 motif-binding surface [34, 35, 40]. Consequently, NS5A has the potential to compete with native binding partners and could act as a functional inhibitor of ASPP2 based on its comparably high affinity for the CTD.

Of note, the C-terminus of NS5A was reported to interact with the HCV Core protein [66]. As ASPP2 also binds to Core we tested whether the Core–NS5A complex may be subject to ASPP2 regulation. Although Core was not identified as an interacting protein of NS5A in our study, we showed that an NS5A PRR peptide could inhibit the ASPP2–Core interaction (Fig. 2e). The consequences of this for HCV biology remain unknown. The ASPP2–Core complex was reported to reduce p53-induced apoptosis [51] in Saos-2 and HepG2 cells, but Huh-7 cells are homozygous for the destabilizing Y220C mutant of p53 [67]. Thus it is unlikely that regulation of p53-mediated apoptosis is a relevant factor in this study.

In this study, we identified a conserved PxϕPxR motif (PRR) in the LCSII region of HCV NS5A that interacts with the SH3 domain of ASPP2. Previous studies showed that mutations of the NS5A PRR motif as well as overexpression of the isolated Bin1 SH3 domain to block interaction with other host proteins had a minimal effect on HCV replication in Huh-7 cells [41, 63, 68, 69], suggesting that the strong ASPP2–NS5A interaction and the regulation of HCV replication are independent. However, another study reported the proline at residue 346 (in gt1a/1b) or 342 (gt2a) to play a role in either RNA replication or assembly of infectious virus respectively in Huh7.5 cells [69]. This proline is likely to be involved in ASPP2–NS5A complex formation, as it is present in the peptides used in Fig. 2d) but missing in the ΔPRR mutant presented in Fig. 2e), hinting towards a potential connection between the ASPP2–NS5A interaction and HCV replication.

Finally, recent sequencing studies identified that Huh7.5 cells acquired additional mutations in p53 and its transcriptional target Bax. As ASPP2 is known to selectively enhance p53’s transcriptional activity towards its pro-apoptotic genes such as Bax, these genetic changes are consistent with the observation that Huh7.5 cells can tolerate higher levels of ASPP2 than that of its parental cell line Huh7. All these suggest that the identified ASPP2–NS5A interaction might mediate ASPP2’s pro-HCV function in Huh-7 cells and that ASPP2 may be a previously unrecognized key host player regulating HCV infection.

## Supplementary Material

Supplementary material 1

Supplementary material 2

Supplementary material 3

Supplementary material 4

## Five reasons to publish your next article with a Microbiology Society journal

1. When you submit to our journals, you are supporting Society activities for your community.
2. Experience a fair, transparent process and critical, constructive review.
3. If you are at a Publish and Read institution, you’ll enjoy the benefits of Open Access across our journal portfolio.
4. Author feedback says our Editors are ‘thorough and fair’ and ‘patient and caring’.
5. Increase your reach and impact and share your research more widely.

Find out more and submit your article at microbiologyresearch.org.

## Abbreviations

CTD: C-terminal domain
HCV: hepatitis C virus
ITC: isothermal titration calorimetry
KD: knock-down
NS: non-structural
OST: One-Strep Tag
RT: room temperature
RT-qPCR: reverse transcription quantitative PCR
siRNA: small interfering RNA
VSV: vesicular stomatitis virus
Y2H: yeast two-hybrid
*β*-ME: *β*-mercaptoethanol
