# Supplementary material for: ASPP2 binds to hepatitis C virus NS5A protein via an SH3 domain/PxxP motif-mediated interaction and potentiates infection
Source: J Gen Virol. Author manuscript; Available in PMC 2024 Mar 5. (PMC7615710; doi:10.1099/jgv.0.001895)
Supplement: Supplementary material 1 [file EMS194310-supplement-Supplementary_material_1.pdf]

Supplementary Figure S1, related to Figure 1

Gene	UniProt entry	Accession	Description	Abundance	Y2H
ZC3H7B	Z3H7B_HUMAN	Q9UGR2	Zinc finger CCCH domain-containing protein 7B	7.785	no
L3MBTL2	A0A0S2Z5X6_HUMAN	A0A0S2Z5X6	L(3)mbt-like 2 (Drosophila)	4.319	no
PIK3AP1	BCAP_HUMAN	Q6ZUJ8	Phosphoinositide 3-kinase adapter protein 1	3.992	no
MLF2	MLF2_HUMAN	Q15773	Myeloid leukemia factor 2	3.338	no
PPP1CC	F8W0W8_HUMAN	F8W0W8	Serine/threonine-protein phosphatase	3.271	no
FOXP4	FOXP4_HUMAN	Q8IVH2	Forkhead box protein P4	2.783	no
KALRN	C9J1B4_HUMAN	C9J1B4	Kalirin (Fragment)	2.77	no
ZNF318	ZN318_HUMAN	Q5VUA4	Zinc finger protein 318	2.679	no
**GRB2**	B0LPF3_HUMAN	B0LPF3	Growth factor receptor-bound protein 2	2.652	**yes**
METAP1	MAP11_HUMAN	P53582	Methionine aminopeptidase 1	2.51	no
DERL2	DERL2_HUMAN	Q9GZP9	Derlin-2	2.494	no
GNAS	Q5JWE9_HUMAN	Q5JWE9	Guanine nucleotide-binding protein G(s) subunit alpha	2.483	no
NOMO3	J3KN36_HUMAN	J3KN36	Nodal modulator 3	2.464	no
ATP6V0C	VATL_HUMAN	P27449	V-type proton ATPase	2.418	no
TMEM245	H7C0G1_HUMAN	H7C0G1	Transmembrane protein 245	2.418	no
TMCC1	TMCC1_HUMAN	O94876	Transmembrane and coiled-coil domains protein 1	2.378	no
CENPH	CENPH_HUMAN	Q9H3R5	Centromere protein H	2.356	no
PHF8	PHF8_HUMAN	Q9UPP1	Histone lysine demethylase PHF8	2.319	no
**TP53BP2**	ASPP2_HUMAN	Q13625	Apoptosis-stimulating of p53 protein 2	2.294	**yes**
YES1	J3QRU1_HUMAN	J3QRU1	Tyrosine-protein kinase	2.289	no

Figure S1 | Supporting Figure 1
Top 20 host interactors of HCV NS5A identified in this study, with two highlighted proteins that were also identified as NS5A binding proteins in the yeast two hybrid (Y2H) assay (de Chassey et al., Mol Syst Biol 2008).

Supplementary Figure S2, related to Figure 2

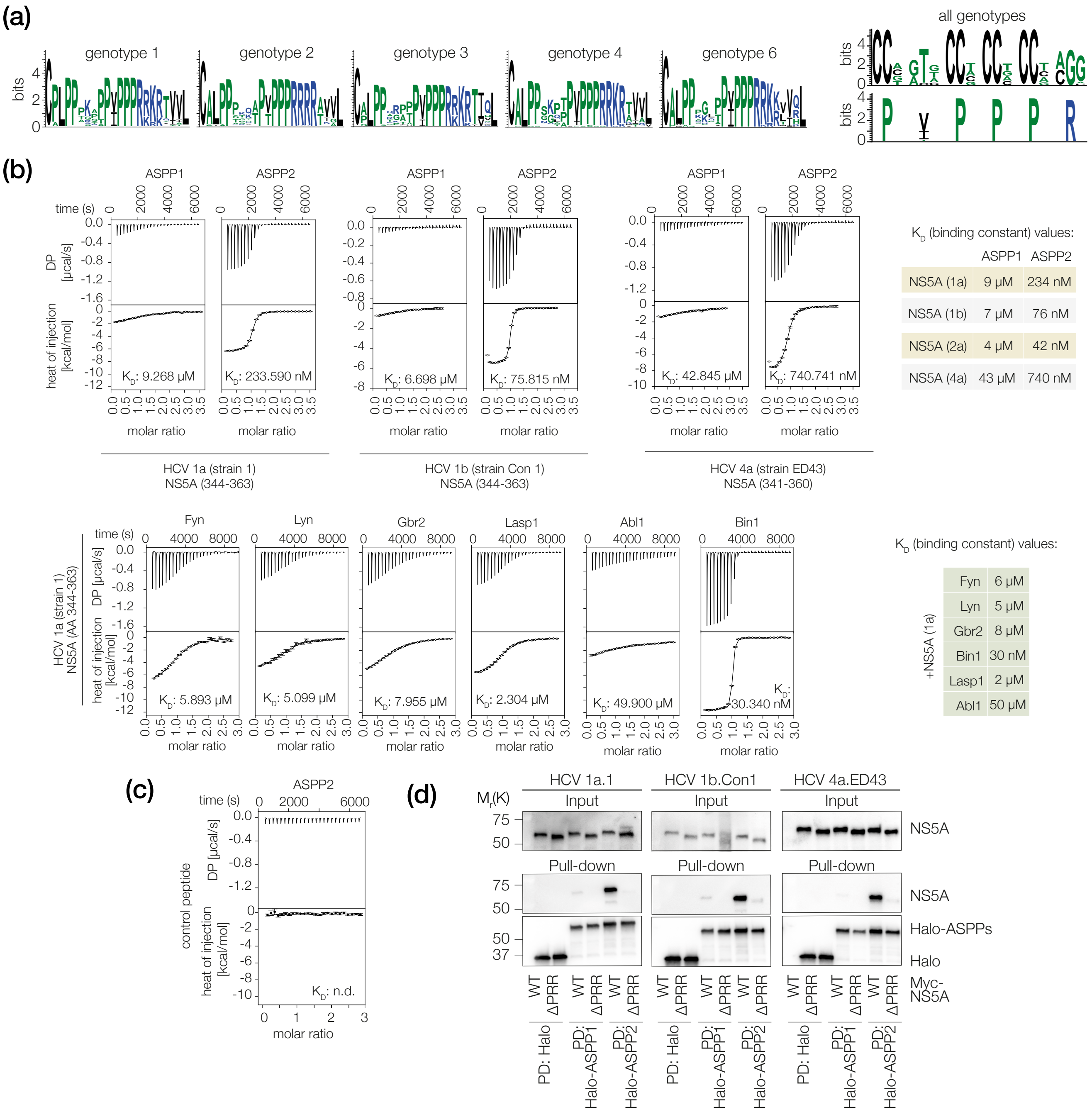

KD (binding constant) values:

	ASPP1	ASPP2
NS5A (1a)	9 µM	234 nM
NS5A (1b)	7 µM	76 nM
NS5A (2a)	4 µM	42 nM
NS5A (4a)	43 µM	740 nM

KD (binding constant) values:

+NS5A (1a)	
Fyn	6 µM
Lyn	5 µM
Gbr2	8 µM
Bin1	30 nM
Lasp1	2 µM
Abl1	50 µM

Figure S2 | Supporting Figure 2

(a) (Top) Consensus sequences of the NS5A PRR for indicated HCV genotypes. The sequence logos were generated by WebLogo (v3) aligning NS5A sequences from 134 different strains (Table S2). (Bottom) Consensus nucleotide and protein sequences of the NS5A PRR for all HCV genotypes.

(b) Isothermal titration calorimetry (ITC) results of the HCV NS5A PRR peptides titrated to ASPP family CTDs (left panel) or known NS5A interactors (right panel). Raw titration profiles are displayed in the top diagrams and integrated heat in the bottom diagram. Best fit of single-site binding model is shown as a solid black line with the resulting KD. Outliers not included in the fit are coloured in grey. All other fit parameters are provided in Table S3.

(c) ITC results of a control peptide lacking a PRR titrated to ASPP2 CTD. Raw titration profiles are displayed in the top diagrams and integrated heat in the bottom diagram. KD could not be determined (n.d.).

(d) Western blot analysis of pull-down assay using immobilized recombinant Halo-tagged ASPP family CTDs to pull down in-vitro translated Myc-tagged NS5A WT and ΔPRR mutants. n = 3 (biological replicates). PD: pull-down; HCV: strains: 1a.1 and 1b.Con ΔPRR: Δ344-363; 4a.ED43 ΔPRR: Δ341-360

Supplementary Figure S3, related to Figure 3

(a)

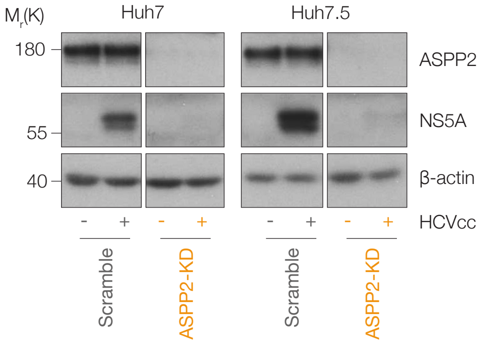

Figure S3 | Supporting Figure 3
(a) Western blot analysis of ASPP2 and NS5A expression in HCVcc infected parental Huh-7 cells after ASPP2-KD. β-actin is shown as a loading control.

Supplementary Figure S4, related to Figure 4

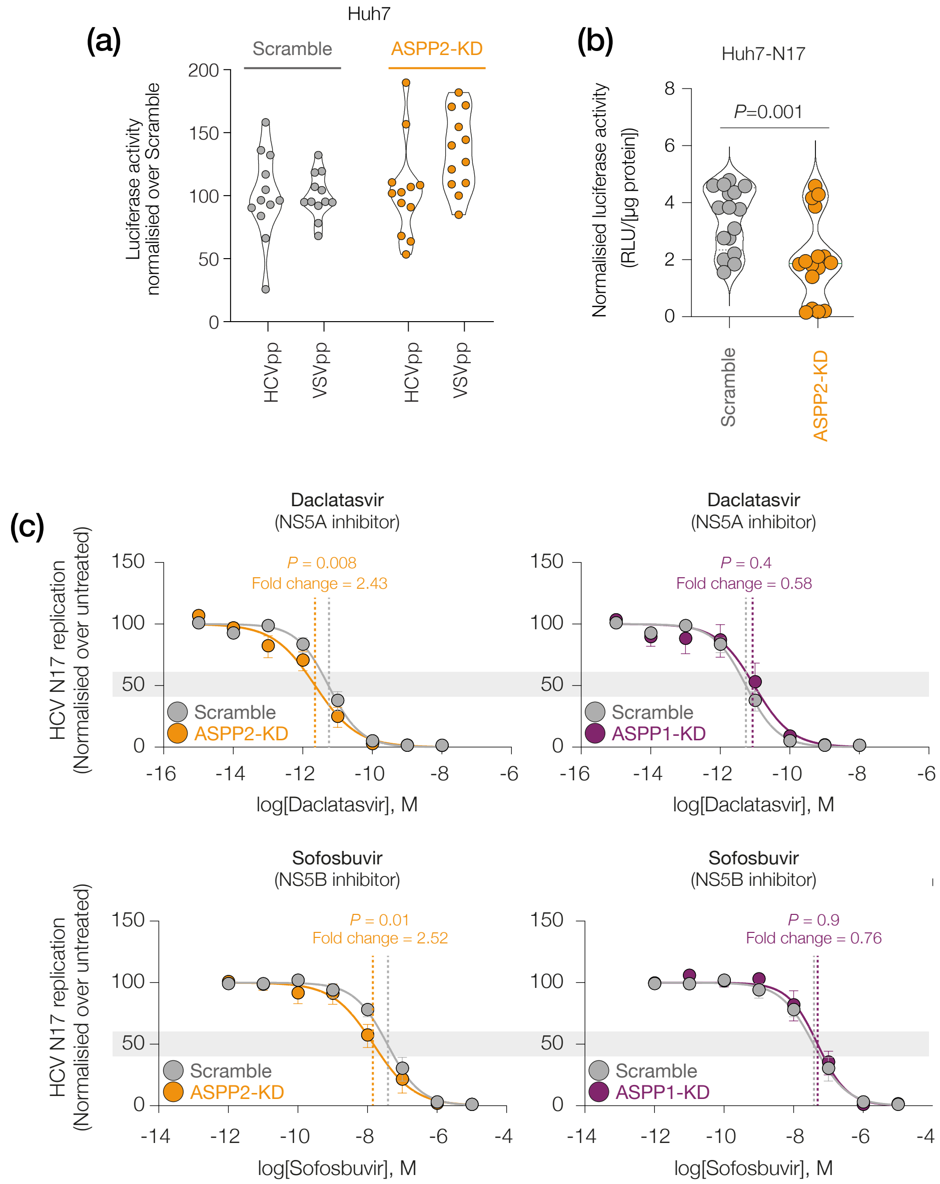

Figure S4 | Supporting Figure 4

(a) Normalised luciferase activity of HCVpp or VSVpp infected Huh-7 cells. Pseudo-particle experiment has been performed infecting Huh-7 cells with 3 different inoculations, with 4 technical replicates per infection. Data presented as mean values normalised over Scramble ± SD.

(b) Luciferase activity in parental Huh7-N17 cells 96 h after ASPP2 knock-down. Data presented as violin plots. Individual values are shown. P by one-way ANOVA test (compared with scramble), n = 5 biological replicates with 4 technical replicates each.

(c) Luciferase activity in replicon Huh7.5-N17 cells subjected to ASPP2 or ASPP1 knock-down for 48 h and subsequently treated with increasing concentrations of indicated antiviral drugs. n = 3-6 biological replicates with 2 technical replicates each.

Supplementary Figure S5, related to Figure 5

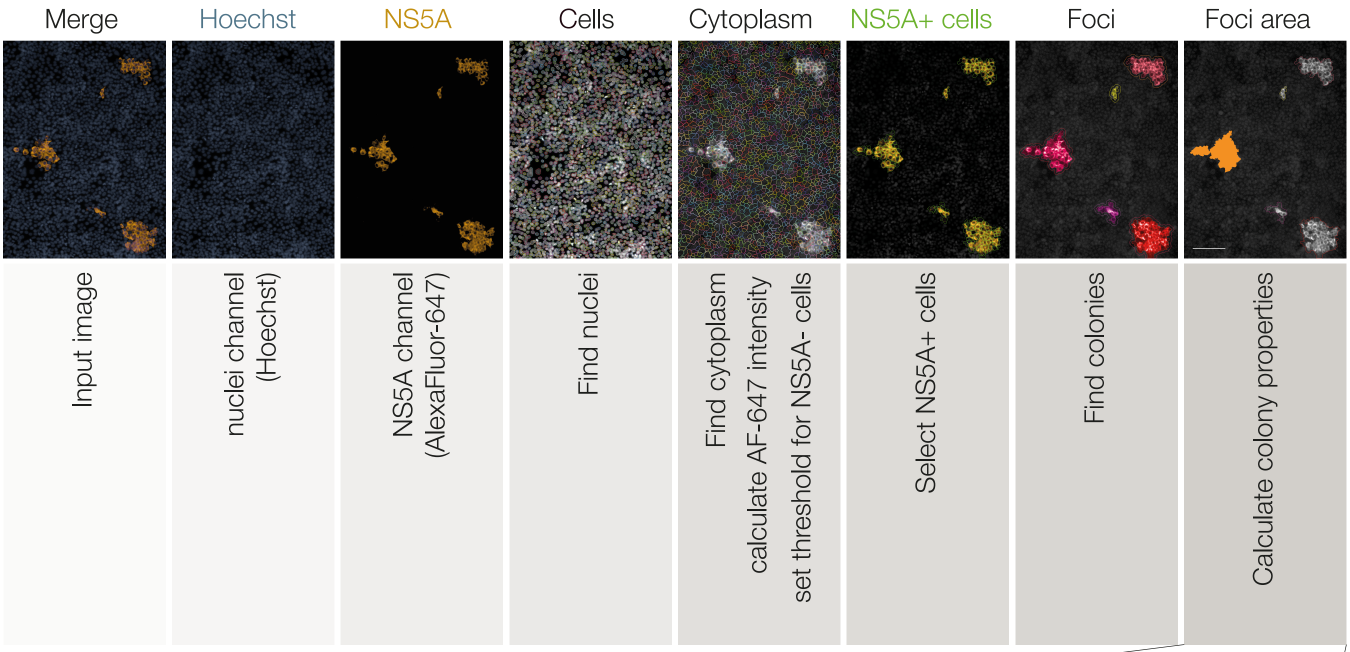

Object No	Colony properties Area [µm²]	Colony properties Roundness	Colony properties Width [µm]	Colony properties Length [µm]
1	13861.2	0.483328	64.1841	200.897
2	1186.75	0.605666	18.8451	63.3565
3	18520.5	0.450368	99.7585	227.873
4	4217.27	0.475906	37.1099	113.428
5	23470.7	0.58795	129.134	197.572

Figure S5 | Supporting Figure 5
Overview of the algorithm to identify, quantify and measure properties of NS5A positive foci of HCV infected cells using Harmony software. Scale bar 250 µm.

Smirnov et al **Table S1** Reagents used in the study		
**Reagent**	**Source**	**Cat no**
**Antibodies**		
NS5A	Charles Rice	9E10
ASPP2	This study	DX54.10
ASPP1	Sigma Aldrich	HPA021603 (RRID:AB_1844384)
β-actin	Santa Cruz	sc-47778 (RRID:AB_626632)
Halo	Promega	G9211 (RRID:AB_2688011)
Mouse immunoglobulins/HRP	Agilent	P0161 (RRID:AB_2687969)
Rabbit immunoglobulins/HRP	Agilent	P0217 (RRID:AB_2728719)
Alexa Fluor 647 anti-mouse Immunoglobulin G	Invitrogen	A-21235 (RRID:AB_2535804)
Myc-tag (clone 4A6)	Merck Millipore	05-724 (RRID:AB_309938)
HA-tag (for WB)	Sigma Aldrich	H3663 (RRID:AB_262051)
HA-tag (for IP)	Bethyl Laboratories	A190-138A (RRID:AB_2631894)
Mouse IgG	Sigma	I5381 (RRID:AB_1163670)
Anti-mouse IgG light chain specific-HRP	Jackson ImmunoResearch	115-035-174 (RRID:AB_2338512)
Anti-mouse HRP	Sigma-Aldrich	A9917 (RRID:AB_258476)
**Bacterial and virus strains**		
HCV virus (strain J6/JFH-1)	(Lindenbach et al., 2005)	n/a
Escherichia coli Rosetta (DE3) Competent Cells	Merck Millipore	70954
Escherichia coli NEB 5-alpha Competent Cells	NEB	C2987
**Chemicals, peptides, and recombinant proteins**		
Daclatasvir	MedChemExpress	HY-10466
Sofosbuvir	MedChemExpress	HY-15005
Puromycin	Sigma Aldrich	P9620
NuPAGE LDS Sample Buffer (4X)	ThermoFisher	NP0008
Benzonase Nuclease	Merck Millipore	70746
HCV [1a.1] NS5A PRR peptide (WLPPPKSPPVPPPRKKRTVVL)	TUCF	custom made
HCV [1b.Con1] NS5A PRR peptide (WLPPAKAPPIPPPRRKRTVVL)	TUCF	custom made
HCV [2a.JFH-1] NS5A PRR peptide (WLPPPKKAPTPPPRRRRTVGL)	TUCF	custom made
HCV [4a.ED43] NS5A PRR peptide (WLPPAKQPPVPSPRRKRTVQL)	TUCF	custom made
**Commercial assays and reagents**		
MycoAlert Kit	Lonza	LT07-118
Bright-Glo Luciferase	Promega	E2610
TnT Quick Coupled Transcription/Translation System T7	Promega	L1170
PEI	Polysciences	23966-1
Lipofectamin 2000	ThermoFisher	11668019
Magne HaloTag Beads	Promega	G7282
**Experimental models: Cell lines**		

Huh7-A	Dr Arvind Patel	n/a
Huh7-B	Dr Michael Wakelam	n/a
Huh7.5-C	Dr Charles Rice	n/a
Huh7.5-D	Dr Peter Simmonds	n/a
Huh7-N17	(Magri *et al.*, 2016)	n/a
Huh7.5-N17	(Magri *et al.*, 2016)	n/a
**Recombinant DNA**		
pNL4.3^{E-R-}	This study	n/a
pcDNA3 c-SRC (WT)	Addgene	42202 (RRID:Addgene_42202)
pENTR4-HaloTag (w876-1)	Addgene	29644 (RRID:Addgene_29644)
HCV NS5A cDNA	ThermoFisher	n/a
SH3 domain cDNA	ThermoFisher	n/a
pET-15b-His10-TEV-ASPP1 CTD (887-1090)	this study	n/a
pET-15b-His10-TEV-ASPP2 CTD (925-1128)	this study	n/a
pGEX-6P-2-His8-TEV-c-Src SH3 (88-144)	this study	n/a
pGEX-6P-2-His8-TEV-Fyn SH3 (85-142)	this study	n/a
pGEX-6P-2-His8-TEV-Lyn SH3 (65-122)	this study	n/a
pGEX-6P-2-His8-TEV-Grb2 SH3 (1-58)	this study	n/a
pGEX-6P-2-His8-TEV-Abl1 SH3 (64–120)	this study	n/a
pGEX-6P-2-His8-TEV-FGR SH3 (110-166)	this study	n/a
pGEX-6P-2-His8-TEV-LASP1 SH3 (205-261)	this study	n/a
pGEX-6P-2-His8-TEV-Bin1 SH3 (513-593)	this study	n/a
pET-15b-His10-Halo-TEV-3xGS	this study	n/a
pET-15b-His10-Halo-TEV-3xGS-ASPP1 CTD (887-1090)	this study	n/a
pET-15b-His10-Halo-TEV-3xGS-ASPP2 CTD (925-1128)	this study	n/a
pcDNA3.1(+)	ThermoFisher	V79020
pcDNA3.1(+)-Myc-HCV [2a.JFH-1] Core	this study	n/a
pcDNA3.1(+)-Myc-HCV [1a.1] NS5A	this study	n/a
pcDNA3.1(+)-Myc-HCV [1a.1] NS5A ΔPRR(344-363)	this study	n/a
pcDNA3.1(+)-Myc-HCV [1b.Con1] NS5A	this study	n/a
pcDNA3.1(+)-Myc-HCV [1b.Con1] NS5A ΔPRR(344-363)	this study	n/a
pcDNA3.1(+)-Myc-HCV [2a.JFH-1] NS5A	this study	n/a
pcDNA3.1(+)-Myc-HCV [2a.JFH-1] NS5A ΔPRR(340-359)	this study	n/a
pcDNA3.1(+)-Myc-HCV [4a.ED43] NS5A	this study	n/a
pcDNA3.1(+)-Myc-HCV [4a.ED43] NS5A ΔPRR(341-360)	this study	n/a
pcDNA3.1(+)-HA -HCV [4a.ED43] NS5A ΔPRR(341-360)	this study	n/a
**Software and algorithms**		
GraphPad 8	Prism	n/a
Clustal Omega	Goujon et al. (2010); Sievers et al. (2011)	https://www.ebi.ac.uk/Tools/msa/clustalo/
NITPIC v1.2.7	Keller et al. (2012); Scheuermann & Brautigam (2015)	https://www.utsouthwestern.edu/labs/mbr/software/

SEDPHAT v15.2b	Houtman et al. (2007)	http://www.analyticalultracentrifugation.com/sedphat/
GUSSI v1.4.2	Brautigam et al. (2015)	https://www.utsouthwestern.edu/labs/mbr/software/

Smirnov et al **Table S4** ITC values

Comp. A (cell)	Comp. B (syringe)	K_D (95% CI)	ΔH (95% CI) [kcal/mol]	IncfA (95% CI)	IncfB (95% CI)
ASPP1 CTD	HCV [1a.1] NS5a PRR peptide	9.268 μM(7.347 to 11.806 μM)	-2.368 (-2.705 to -2.160)	0.039 (<0 to 0.121)	0 (fixed)
ASPP2 CTD	HCV [1a.1] NS5a PRR peptide	233.590 nM (207.910 to 261.638 nM)	-6.327 (-6.385 to -6.269)	0.003 (<0 to 0.008)	0 (fixed)
Fyn SH3	HCV [1a.1] NS5a PRR peptide	5.893 μM (3.090 to 4.920 μM)	-7.765 (-8.342 to -7.301)	0.046 (0.010 to 0.087)	0 (fixed)
Lyn SH3	HCV [1a.1] NS5a PRR peptide	5.099 μM (3.870 to 6.648 μM)	-5.522 (-5.948 to -5.205)	0.033 (<0 to 0.099)	0 (fixed)
Grb2 SH3	HCV [1a.1] NS5a PRR peptide	7.955 μM (7.161 to 8.866 μM)	-6.976 (-7.309 to -6.681)	0.094(0.076 to 0.112)	0 (fixed)
Bin1 SH3	HCV [1a.1] NS5a PRR peptide	30.340 nM (21.367 to 41.246 nM)	-11.497 (-11.622 to -11.372)	0.026 (0.021 to 0.031)	0 (fixed)
Lasp1 SH3	HCV [1a.1] NS5a PRR peptide	2.304 μM (2.083 to 2.546 μM)	-6.048 (-6.176 to -5.927)	0.044 (0.031 to 0.058)	0 (fixed)
Abl1 SH3	HCV [1a.1] NS5a PRR peptide	49.900 μM (46.220 to 53.955 μM)	-8.163 (-8.443 to -7.902)	0 (fixed)	0 (fixed)
ASPP1 CTD	HCV [1b.Con1] NS5A PRR peptide	6.698 μM (4.801 to 9.426 μM)	-0.959 (-1.139 to -0.841)	0.102 (0.017 to 0.204)	0 (fixed)
ASPP2 CTD	HCV [1b.Con1] NS5A PRR peptide	75.815 nM (63.38907 to 89.95193 nM)	-5.429 (-5.491 to -5.368)	0.049 (0.044 to 0.055)	0 (fixed)
ASPP1 CTD	HCV [2a.JFH-1] NS5A PRR peptide	3.778 μM (3.583 to 3.983 μM)	-5.190 (-5.280 to -5.105)	0.083 (0.071 to 0.096)	0 (fixed)
ASPP2 CTD	HCV [2a.JFH-1] NS5A PRR peptide	42.355 nM (35.704 to 49.579 nM)	-10.445 (-10.512 to -10.371)	0.0818 (0.0785 to 0.0852)	0 (fixed)
ASPP1 CTD	HCV [4a.ED43] NS5A PRR peptide	42.845 μM (24.540 to 52.005 μM)	-3.809 (-4.099 to -3.406)	0 (fixed)	0.031 (<0 to 0.225)
ASPP2 CTD	HCV [4a.ED43] NS5A PRR peptide	740.741 nM (664.840 to 823.592 nM)	-8.001 (-8.109 to -7.895)	0.134 (0.125 to 0.143)	0 (fixed)
